# Association of Renal Elasticity and Renal Function Progression in Patients with Chronic Kidney Disease Evaluated by Real-Time Ultrasound Elastography

**DOI:** 10.1038/srep43303

**Published:** 2017-02-27

**Authors:** Hugo You-Hsien Lin, Yu-Li Lee, Kun-Der Lin, Yi-Wen Chiu, Shyi-Jang Shin, Shang-Jyh Hwang, Hung-Chun Chen, Chi-Chih Hung

**Affiliations:** 1 Division of Nephrology, Department of Internal Medicine, Kaohsiung Medical University Hospital, Kaohsiung Medical University, Kaohsiung, Taiwan; 2 Department of Internal Medicine, Kaohsiung Municipal Ta-Tung Hospital, Kaohsiung Medical University, Kaohsiung, Taiwan; 3 Graduate Institute of Medicine, College of Medicine, Kaohsiung Medical University, Kaohsiung, Taiwan; 4 Lipid Science and Aging Research Center, Kaohsiung Medical University, Kaohsiung, Taiwan; 5 Department of Physiology & Biophysics, University of California at Irvine, Irvine, CA 92697, USA; 6 Sue and Bill Gross Stem Cell Research Center, University of California at Irvine, Irvine, CA 92697, USA; 7 Endocrinology and Metabolism, Department of Internal Medicine, Kaohsiung Medical University Hospital, Kaohsiung Medical University, Kaohsiung, Taiwan.

## Abstract

Glomerulosclerosis and tubulointerstitial fibrosis are associated with lower renal parenchymal elasticity. This study was designed to evaluate the predictive ability of renal elasticity in patients with chronic kidney disease (CKD). 148 non-CKD patients and 227 patients with CKD were recruited. 145 (38.7%) were female, 166 (73.1%) had diabetes, the mean estimated glomerular filtration rate (eGFR) was 33.9 ± 15.8 ml/min/1.73 m^2^ and the median urinary protein-to-creatinine ratio (UPCR) 502 (122–1491) mg/g. Patients with later stages of CKD had lower renal elasticity values, indicating stiffer kidneys (p < 0.001), and smaller kidney (p < 0.001). Renal elasticity correlated with log-transformed UPCR (β = −7.544, *P* < 0.001). Renal length correlated with age (β = −0.231, *P* < 0.001), sex (β = −3.730, *P* < 0.001), serum albumin level (β = −3.024, *P* = 0.001), body mass index (β = 0.390, *P* = 0.009) and eGFR (β = 0.146, *P* < 0.001). In fully-adjusted logistic regression model, the odds ratio (OR) per 10 unit change in renal elasticity for rapid renal deterioration was 0.928 (95% CI, 0.864–0.997; *P* = 0.042). The OR per 1 mm change in renal length for rapid renal deterioration was 1.022 (95% CI, 0.994–1.050; *P* = 0.125). Renal elasticity is associated with proteinuria and rapid renal deterioration in patients with CKD.

Chronic kidney disease (CKD) is becoming a global public health problem^1^. It can lead to serious sequela, including end-stage renal disease (ESRD) and cardiovascular (CV) disease and mortality^2^. At present, kidney diseases are detected clinically by decreases in estimated glomerular filtration rate (eGFR) and increases in proteinuria. One of the most popular non-invasive imaging techniques used to evaluate kidney diseases is traditional renal sonography which measures renal length, cortical thickness, and cortical echogenicity^3^. While renal length and cortical thickness have been significantly correlated with eGFR^3^,^4^, these parameters represent late-stage changes requiring more aggressive treatment when detected. Therefore, there is a need for other noninvasive clinical imaging tools capable of detecting kidney diseases at earlier stages.

While pathological findings of glomerulosclerosis and tubulointerstitial fibrosis obtained through biopsies have been associated with worse outcomes in renal diseases^5^,^6^,^7^, many noninvasive methods, including serum and urinary biomarker measurements and renal image studies, have been used to assess these renal injuries in humans^8^,^9^,^10^. There has also been research into evaluating renal injury using the ultrasound elastography, which has been used to measure kidney elasticity, in patients with renal transplant^11^. In fact, several imaging techniques have been used to assess the degree of renal damage in patients with different kidney diseases. They include transient elastography (TE), acoustic radiation force impulse imaging (ARFI) and shear wave elastography (SWE), which measure the speed of the shear wave generated by an acoustic pulse or a vibration through the tissue, as well as real-time elastography (RTE), which assesses elasticity based on the physical strain within the tissue created by external compression^12^,^13^,^14^,^15^. However, there are very few studies investigating the use of RTE to evaluate kidney status in patients with CKD and its predictive value.

Thus, this prospective study was designed to investigate the possible correlation of RTE-measured renal elasticity as well as traditional echography measurements of kidney size and CKD stage with progression of renal dysfunction in patients with and without CKD. Changes in kidney function (EGFR) were also followed for six months.

## Methods

### Participants and Measurements

This prospective observational study was performed in southern Taiwan from October 2011 to November 2013. We included 149 non-CKD patients and 230 patients with CKD Stages 3 to 5 assessed using RTE from the outpatient department at Kaohsiung Municipal Ta-Tung Hospital. We excluded one patient in the non-CKD group and three patients in CKD group because they had abnormal renal image findings (renal tumor and hydronephrosis) detected using conventional sonography. The patients were followed until December 31, 2014. The protocol for this study was approved by the Institutional Review Board of Kaohsiung Medical University Hospital (KMUH-IRB-20110225), which operates Kaohsiung Municipal Ta-Tung Hospital. All participants provided written informed consent. All methods were performed in accordance with the relevant guidelines and regulations.

From the medical charts of the patients, we obtained baseline patient characteristic data, including demographic features (age, gender), comorbidities (Diabetes Mellitus [DM] and hypertension), medication history (anti-hypertension agents and oral hypoglycemic agents), lifestyle, physical examination findings (body mass index [BMI], mean blood pressure [MBP]) and laboratory data (hemoglobin, albumin, blood glucose, cholesterol, triglyceride, glycated hemoglobin, potassium, bicarbonate and uric acid). Demographic and initial laboratory data were collected at first visit. Follow-up laboratory data and the medical history were collected by chart review. Hypertension was defined based on clinical diagnosis and medication prescribed.

### Measurement of renal length, width, cortical thickness, and elasticity by sonography and real-time elastography

RTE was performed using a Hitachi HIVISION PREVIUS sonographer with elastrography (HitachiMedical, Tokyo, Japan) and a linear probe (EUP-C715; central frequency, 1–5 MHz). The linear probe was placed on both sides of the kidney with the patient lying supine and both arms elevated above the head. A single board-certified nephrologist with more than ten years of sonography experience performed all RTE and traditional ultrasound for this study, eliminating inter-observer bias. Traditional ultrasound variables recorded in this study were length, width, and cortical thickness.

Tissue elasticity distribution was assessed in RTE by recording response to strain and stress within the regions of interest (ROI) (Supplement Fig. 1). In all participants, a rectangular area (15 mm long and 10 mm wide)within the renal cortex area was chosen as the ROI. The equipment automatically adjusted for internal distortion in the kidney tissue caused by the beating of heart. To obtain better images, we first scanned each participant to adjust our approach to the ROI to avoid artifacts caused by renal cysts and attenuation by the liver. Numerical strain values for the pixels were converted into a color image of the rectangular area. The color images ranged from red (minimum strain starting from 0) to orange, yellow, green, and blue (maximum strain 255). A histogram was generated based on relative strain value expressed as mean (MEAN) plus standard deviation (SD).

### Renal function progression

Renal function was assessed using estimated glomerular filtration rate (eGFR) as definedby the simplified Modification of Diet in Renal Disease (MDRD) Study equation^16^, following the formula: eGFRmL/min/1.73 m^2^ = (186) × (Serum creatinine^−1.154^) × (Age^−0.203^) (C) with C being 0.742 for women, 1.212 for black patients, or 1 for all others. To assess the renal outcome in patients with CKD Stages 3a to 5 in a limited observation period, we defined renal function progression bye GFR slope. Rapid renal function deterioration was defined when eGFR slope <−5 ml/min/1.73 m^2^/year.

### Statistical Analysis

Baseline characteristics were analyzed descriptively as counts and percentages for the categorical data and as means with standard deviation (SD) and medians with interquartile ranges (IQR) for continuous variables with near normal distributions. Multivariate linear regression analysis was used to evaluate the relationship between renal elasticity and cross-sectional parameters. Multivariate logistic regression analysis was used to evaluate the relationship between renal elasticity and rapid renal function progression. Covariates were selected based on a review of the literature and our previous publications. Continuous variables with skewed distributions were log-transformed to obtain normal distributions^17^. The model was adjusted for age, gender, diabetes, eGFR, log-transformed UPCR, hemoglobin, albumin, and BMI. The intra-observer coefficient of variation (CV) (SD/mean × 100%) for repeat RTE value measurements was calculated. A *p-*value of <0·05 was considered significant. All statistical operations was performed using the R 2·15·2 software (R Foundation for Statistical Computing, Vienna, Austria) and Statistical Package for Social Sciences version 18·0 for Windows (SPSS Inc., Chicago, IL).

## Results

### Baseline characteristics of the subjects

In total, this study included 148 non-CKD patients and 227 patients with CKD. In the cohort, mean age was 63.2 ± 15.4 year-old and 145 (38.7%) participants were female (Table 1).One hundred three (27.5%) participants had hypertension and 223 (59.5%) had diabetes. Patients with CKD stages 3 to 5 had a significant decrease in serum hemoglobin, high density lipoprotein cholesterol, albumin, sodium bicarbonate and increase of serum uric acid and potassium, compared to non-CKD patients(p for trend all <0.01). After an average follow-up period of 517 (180–1126)days, the total cohort was found to have an eGFR slope of–0.5 (−4.6 to 0.4) ml/min/1.73 m^2^/yr.

### Comparison of renal image measurements among non-CKD patients and patients with CKD

**Figure 1:**
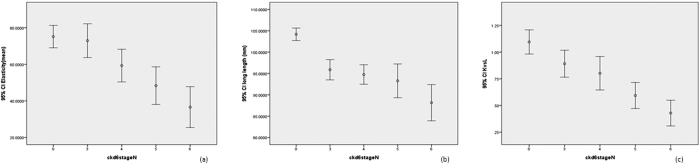
Box plots of (**a**) real time elastography for non-CKD patients and patients with CKD stage 3a to 5; (**b**) renal length; (**c**) kidney-to-liver elasticity ratio.

**Figure 2:**
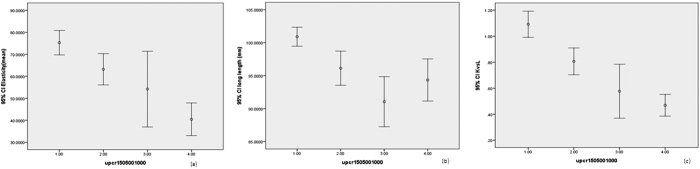
Box plots of (**a**) real time elastography for non-CKD patients and patients with UPCR < 150, 150–500, 500–1000 and >1000 mg/g; (**b**) renal length; (**c**) kidney-to-liver elasticity ratio.

The renal elasticities were 75.1 ± 37.8. 72.9 ± 37.6, 59.3 ± 40.3, 48.3 ± 33.8, and 36.6 ± 33.0 for non-CKD patients and patients with CKD stages 3a, 3b, 4 and 5, respectively (p for trend <0.001) (Table 1) (Fig. 1a). There is a significant decreasing trend was found in kidney-to-liver elasticity ratio (1.1 ± 0.7, 0.9 ± 0.5, 0.8 ± 0.7, 0.6 ± 0.4, 0.4 ± 0.4, respectively) (p for trend <0.001), renal long length (104.2 ± 9.0, 95.9 ± 9.7, 94.8 ± 10.3, 93.3 ± 13.0, 88.2 ± 12.5, respectively) (p for trend <0.001) and renal cortex thickness (9.7 ± 5.0, 8.8 ± 2.4, 8.6 ± 2.2, 8.5 ± 2.0, 8.4 ± 3.1, respectively) (p = 0.003) among non-CKD patients and patients with CKD stages 3 to 5 (Table 1) (Fig. 1b,c). A significant difference was found in renal elasticity (64.5 ± 39.5, 75.3 ± 39.4, 63.2 ± 31.3, 54.2 ± 47.8, 40.3 ± 31.2, respectively) (p < 0.001), renal long length (97.9 ± 11.6, 100.9 ± 10.3, 96.1 ± 11.4, 91.1 ± 10.5, 94.3 ± 13.4, respectively) (p < 0.001) and kidney-to-liver elasticity ratio (4.4 ± 5.4, 3.7 ± 5.4, 4.6 ± 5.3, 6.8 ± 5.9, 5.1 ± 5.3, respectively) (p = 0.012) among non-CKD patients and patients with UPCR < 150, 150–500, 500–1000 and >1000 mg/g (Fig. 2a,b,c).

### Factors related to renal elasticity and renal length

As can be seen in Table 2, the results of our multivariate linear regression analysis, renal elasticity only correlated with log-transformed UPCR (β = −7.544, *P* < 0.001) (Table 2) in all three models. Renal length correlated positively with BMI, male (vs. female) and eGFR and it correlated negatively with age and serum albumin. Kidney-to-liver elasticity ratio correlated negatively with diabetes and log-transformed UPCR.

### Renal elasticity, renal length, and rapid renal progression

In our fully-adjusted logistic regression model (Table 3), the odds ratio (OR) of renal elasticity (per 10 unit) for rapid renal deterioration was 0.928 (95% CI, 0.864–0.997; *P* = 0.042), long renal length (per 1 mm) 1.022 (95% CI, 0.994–1.050; *P* = 0.125), and kidney-to-liver elasticity ratio (per 10%) 0.942 (95% CI, 0.894–0.992; *P* = 0.030).

### Subgroup analysis of renal images in non-CKD patients and patients with CKD

**Figure 3:**
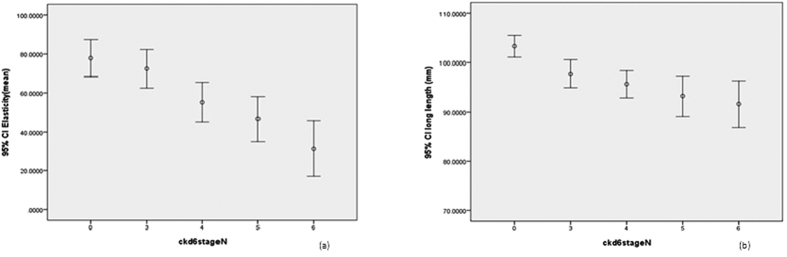
Box plots of (**a**) real time elastography for non-CKD patients and patients with diabetic CKD stage 3a to 5; (**b**) renal length.

**Figure 4:**
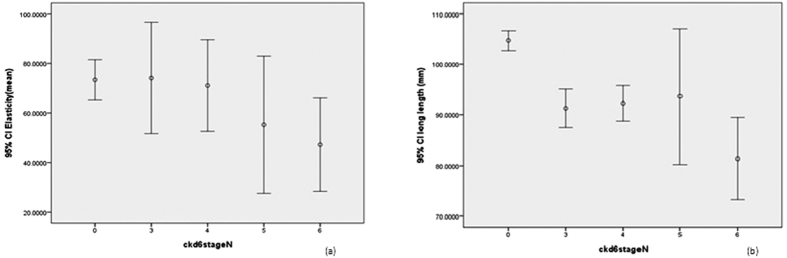
Box plots of (**a**) real time elastography for non-CKD patients and patients with non-DM CKD stage 3a to 5; (**b**) renal length.

**Figure 5:**
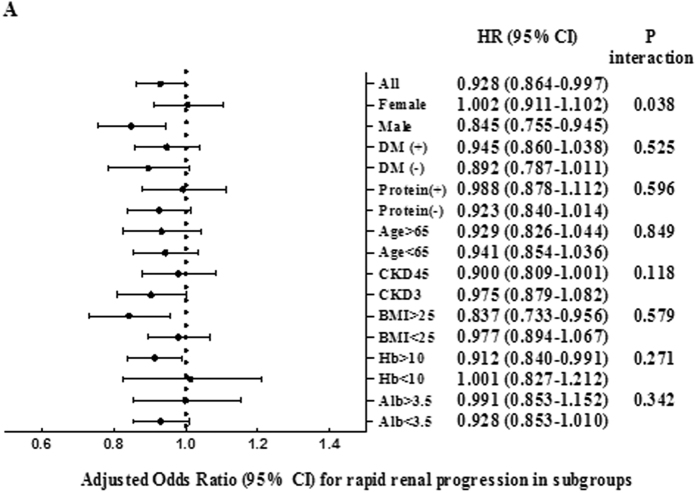
Foresttree plot of the OR per 1 unit increase of elasticity for rapid renal progression.

Because patients with diabetes have larger kidneys, we studied renal elasticity and renal length in patients with and without diabetes. In patients with diabetes, there were no significant differences in renal elasticity (77.9 ± 36.1, 72.4 ± 33.8, 55.2 ± 39.8, 46.6 ± 33.6, 31.3 ± 33.8, respectively) (p = 0.700) and renal length (103.3 ± 8.4, 97.7 ± 9.8, 95.6 ± 11.0, 93.2 ± 11.9, 91.5 ± 11.2, respectively) (p = 0.194) among non-CKD patients and patients with CKD stages 3 to 5 (Fig. 3a,b). In patients without diabetes, there were significant differences of renal length (104.7 ± 9.4, 91.3 ± 8.0, 92.3 ± 7.8, 93.6 ± 17.4, 81.4 ± 12.7, respectively) (p < 0.001) (Fig. 4a,b). Logistic regression analysis for rapid renal progression was performed in pre-specified subgroups. A significant interaction was observed in the subgroup divided by gender (*p* = 0.038) (Fig. 5).

The RTE intra-observer mean CV was 11% (range 4–19%).

## Discussion

This prospective study found renal elasticity measured by RTE to be correlated with proteinuria (p < 0.001) and renal length to be correlated positively with BMI (p = 0.009), sex (p < 0.001) and eGFR (p < 0.001) and negatively with age (p < 0.001) and serum albumin (p = 0.001). We found a significant association between rapid renal deterioration and renal elasticity (p = 0.042) but not renal length(p = 0.125) in patients with CKD Stages 3–5 assessed by traditional sonography. Together these findings suggest that renal elasticity measured by RTE correlated better than traditional echography with CKD stage and could effectively predict progression of renal dysfunction.

Renal size measured by renal sonography is well-known to be correlated with eGFR. Yamashita *et al*.^3^ reported renal cortical thickness, but not bipolar length or parenchymal thickness, to be correlated with eGFR. Belandet *et al*.^4^ reported renal cortical thickness to be more closely related to eGFR than renal length. In these two studies, however, only single variate analysis without considering other factors such as proteinuria and diabetes was performed. The normal ranges of renal length and cortical thickness is wide due to individual differences and difference in operator measurements.

In kidney disease, a decrease in renal size is a late and irreversible finding, and as such it may not be a practical prognostic parameter. Other physical parameters of renal parenchyma besides renal size can be measured. Increased renal echogenicity has been observed by renal ultrasonography before findings of decreased renal size^9^. However, renal echogenicity cannot easily be standardized or digitalized. Another physical parameter, kidney stiffness, can be measured by elastography using quasi-static or dynamic method^12^,^13^. In the quasi-static method, images are obtained by applying a stimulus to induce tissue strain and rebound is measured in terms of length changes. In the dynamic method, images are obtained by detecting and tracking shear waves after mechanical vibration of tissue^18^. Kidney stiffness can also be measured by Fibroscan with transient elastography. This method has been used in the evaluation of liver disease and renal allografts^12^,^19^,^20^. However, the application of this method is impractical for patients with CKD because it only measures elasticity, but not B-mode, which can be provided by traditional sonography. Consequently, Fibroscan may produce unreliable results, with elasticity values exceeding 15% error in quantification^21^. Another physical parameter, physical strain (displacement) measured real-time by RTE can easily capture the elasticity value after the most appropriate region of interest (ROI) has been located.RTE captures 2D strain images induced by internal heart beats which can be used to indicate increasing patchiness that occurs with worsening organ fibrosis^22^.

Previous studies have compared the measurement of renal elasticity with renal biopsy, which is the most accepted method of evaluating kidney status. One study, using RTE confirmed by ultrasound-guided renal biopsy to study renal fibrosis, found their non-fibrosis and fibrosis groups to have significantly different renal elasticity results, though no significant difference was observed between their mild fibrosis and moderate fibrosis groups^23^. Another study used Fibroscanas well as ultrasound-guided renal biopsy to evaluate renal stiffness in patients with and without renal interstitial fibrosis and reported renal stiffness to be correlated with degree of renal interstitial fibrosis (p < 0.05)^24^. Oriacchio *et al*. found a significant correlation between renal elasticity measured by RTE with degree of renal fibrosis in patients receiving renal transplants^13^. Together, these studies suggest that elastography may be feasibly and effectively used as a noninvasive tool for predicting renal fibrosis.

Renal elasticity has also been evaluated in cross-sectional studies of CKD. One study of children with vesicoureteral reflux found the renal elasticity in the affected kidneys to be significantly different from healthy kidneys^25^. Another study of patients with CKD discovered a correlation between renal elasticity and eGFR^26^. A study of 319 patients with CKD found renal elasticity, which was represented as shear wave velocity (SWV) measured by ARFI, decreased concomitantly with a decline in eGFR^27^. However, in a study of 45 patients with CKD, SWV did not significantly differ among the CKD stages^28^. The current investigation found renal elasticity worsened progressively from CKD Stage 3 to 5.Our multivariate analysis, however, revealed that renal elasticity was only associated with proteinuria, after adjusting for various factors. We believe that the correlation between elasticity and proteinuria may be a possible link between renal elasticity and early renal fibrosis. The presence of proteinuria is characterized with infiltration of inflammatory cells into the renal interstitium and replacement of the tubulointerstitium by fibrous scar^29^.

Diabetic nephropathy (DN) is the leading cause of CKD^30^. In traditional sonography, DN is characterized initially by increases in renal length and renal parenchymal thickness. However, when DN deteriorates clinically, renal length might remain within the normal range and thus there would be no specific sign of DN in a sonographic evaluation. Several studies have tried to evaluate DN using different sonographic measurements. In a study by Kim *et al*.^31^, the resistive index (RI) was high when it was evaluated in the arcuate and interlobar arteries of the kidney. In a study of Jeong *et al*.^32^, the renal venous impedence index was low in patients with DN. The diagnostic and prognostic value of these vascular parameters remains unclear. There are few studies evaluating renal elasticity in patients with DN. One by Goya *et al*.^33^ found healthy patients to have significantly differentrenal elasticities than patients with different stages of diabetic nephropathy. The present study observed a significantly decreasing trend in renal elasticity when comparing diabetes patients without CKD with diabetes patients with CKD stages 3–5 (p < 0.001).Therefore, an increase in renal parenchymal stiffness may function as a surrogate indicator of DN.

A few studies have evaluated the association between renal elasticity and renal outcomes clinically. Tatar *et al*.^34^, evaluating renal elasticity in patients with renal transplants, found that renal elasticity could predict graft outcome (p < 0.05). Gao *et al*.^35^ reported that renal elasticity could distinguish severity level of renal cortical fibrosis, a good predictor for renal outcome. Several studies^12^,^13^,^36^, evaluating renal elasticity in renal transplantation patients receiving protocol biopsies, have reported renal elasticity to be significantly correlated with renal fibrosis. There is still another study, Asano *et al*.^27^, finding that SW elastography values measured by ARFI to be confounded by diminished blood flow. Together, these results may explain in part the reason that renal elasticity can predict progression of kidney disease. This study is the first to demonstrate that renal elasticity measured by RTE, not renal length, can predict rapid renal function deterioration in patients with CKD.

### Limitations

This study has several limitations. First, there are several potential factors may influence the accuracy of different renal strain measurements, including changes in the depth of ROI, geometry of acquisition, and subcutaneous fat thickness. Second, we did not measure renal blood flow and resistance index, which might have contributed to renal elasticity in our subjects. Third, there are several clinical parameters such as blood pressure and cardiovascular disease were not recorded. Fourth, rapid renal progression is a surrogate endpoint, not a hard outcome.

## Conclusion

In summary, renal elasticity measured by RTE with renal sonography correlates well with proteinuria and rapid renal deterioration in patients with CKD. This objective noninvasive method can be used to monitor patients with CKD. Whether it can be used as a predictor for ESRD is another matter and should be studied in the near future.

## Additional Information

**How to cite this article:** You-Hsien Lin, H. *et al*. Association of Renal Elasticity and Renal Function Progression in Patients with Chronic Kidney Disease Evaluated by Real-Time Ultrasound Elastography. *Sci. Rep.* **7**, 43303; doi: 10.1038/srep43303 (2017).

**Publisher's note:** Springer Nature remains neutral with regard to jurisdictional claims in published maps and institutional affiliations.

## Supplementary Material

Supplementary Figure

## Figures and Tables

**Table 1:** Patient characteristics by CKD stage.

Variable	All	Non-CKD	CKD stage 3a	CKD stage 3b	CKD stage 4	CKD stage 5	*P* for trend
No. of patients	375	148	66	81	44	36	—
Age (yr)	63.2 ± 15.4	53.3 ± 15.0	70.2 ± 10.0	70.6 ± 12.4	71.2 ± 12.4	64.8 ± 12.5	0.312
Gender (female)	145 (38.7%)	61 (41.2%)	22 (33.3%)	22 (27.2%)	22 (50.0%)	18 (50.0%)	0.940
Height (cm)	162.5 ± 8.6	164.0 ± 8.6	162.0 ± 7.4	162.8 ± 7.5	158.8 ± 10.3	160.6 ± 9.0	0.042
Body weight (Kg)	64.8 ± 11.7	63.4 ± 11.2	65.9 ± 11.5	66.9 ± 11.0	65.3 ± 10.9	62.5 ± 16.1	0.263
BMI (Kg/m^2^)	24.4 ± 3.4	23.5 ± 3.5	25.0 ± 3.4	25.2 ± 3.5	25.8 ± 3.4	23.9 ± 4.1	0.159
Diabetes Mellitus	223 (59.5%)	57 (38.5%)	47 (71.2%)	60 (74.1%)	35 (79.5%)	24 (66.7%)	0.105
Hypertension	103 (27.5%)	18 (12.2%)	19 (28.8%)	29 (35.8%)	19 (43.2%)	18 (50.0%)	<0.001
**Renal function**							
eGFR (ml/min/1.73 m^2^)	58.8 ± 36.7	97.0 ± 24.7	50.7 ± 5.9	38.3 ± 3.9	21.9 ± 4.2	7.7 ± 3.7	<0.001
UPCR (mg/gm)	137 (40–687)	30 (30–66)	91 (44–209)	403 (101–1123)	948 (526–3951)	2063 (1186–4437)	< 0.001
**Renal Image**							
Renal elasticity	64.5 ± 39.5	75.1 ± 37.8	72.9 ± 37.6	59.3 ± 40.3	48.3 ± 33.8	36.6 ± 33.0	<0.001
Liver elasticity	80.9 ± 24.1	75.2 ± 26.1	85.9 ± 19.8	79.9 ± 23.2	85.6 ± 20.9	87.4 ± 22.2	0.036
Kidney to liver elasticity ratio	0.9 ± 0.7	1.1 ± 0.7	0.9 ± 0.5	0.8 ± 0.7	0.6 ± 0.4	0.4 ± 0.4	<0.001
Renal length (mm)	97.9 ± 11.6	104.2 ± 9.0	95.9 ± 9.7	94.8 ± 10.3	93.3 ± 13.0	88.2 ± 12.5	<0.001
Renal width (mm)	45.6 ± 6.4	45.4 ± 6.5	46.2 ± 6.0	46.3 ± 5.7	45.2 ± 7.0	43.9 ± 7.5	0.779
Renal cortex thickness (mm)	9.0 ± 3.7	9.7 ± 5.0	8.8 ± 2.4	8.6 ± 2.2	8.5 ± 2.0	8.4 ± 3.1	0.003
**Laboratory data**							
Hemoglobin (g/dl)	12.6 ± 2.4	13.9 ± 1.8	12.8 ± 2.1	12.4 ± 2.0	11.3 ± 2.0	8.9 ± 1.1	<0.001
Sugar (AC) (mg/dl)	158.9 ± 93.4	147.5 ± 85.1	144.4 ± 65.8	175.3 ± 115.9	178.0 ± 113.0	171.6 ± 78.0	0.018
HbA1c (%)	7.1 ± 2.0	7.2 ± 2.2	6.9 ± 1.6	7.4 ± 2.0	7.1 ± 2.3	6.6 ± 1.3	0.424
Total cholesterol (mg/dl)	188 (158–219)	197 (155–219)	180 (145–214)	194 (153–232)	160 (117–234)	207 (148–226)	0.237
Triglyceride (mg/dl)	127 (78–179)	106 (63–182)	95 (59–147)	137 (82–191)	132 (61–194)	151 (113–205)	0.012
Uric acid (mg/dl)	6.8 ± 2.3	5.8 ± 2.2	7.0 ± 2.5	7.2 ± 1.9	7.9 ± 1.8	8.2 ± 2.0	<0.001
Albumin (g/dl)	4.1 ± 0.6	4.3 ± 0.6	4.0 ± 0.6	4.0 ± 0.6	3.9 ± 0.6	3.8 ± 0.6	<0.001
K (mEq/l)	3.9 ± 0.7	3.7 ± 0.6	3.9 ± 0.6	4.1 ± 0.6	4.3 ± 0.7	4.4 ± 0.7	<0.001
HCO3 (mEq/l)	23.5 ± 5.3	24.8 ± 5.4	23.4 ± 4.6	23.7 ± 5.5	21.4 ± 4.8	20.2 ± 4.2	<0.001
**Outcome**							
Follow-up days	517 (180–1126)	594 (180–1156)	425 (178–905)	640 (204–1080)	548 (204–981)	510 (182–974)	0.315
GFR slope (ml/min/1.73 m^2^/yr)	−0.5 (−4.6 to 0.4)	0.0 (−0.9 to 2.7)	0.0 (−6.9 to 0.9)	−2.6 (−10.4 to 0.0)	−0.5 (−19.3 to 12.3)	−3.5 (−9.2 to 0.2)	<0.001
Rapid renal function progression	89 (23.7%)	28 (18.9%)	10 (15.2%)	20 (24.7%)	18 (40.9%)	13 (36.1%)	0.006

CKD: chronic kidney disease; BMI: body mass index; eGFR: estimated glomerular filtration rate; HbA1C: glycated haemoglobin; K: potassium; HCO3: bicarbonate.

Data are presented as mean ± standard error, median (interquartile range), or count (percentage).

**Table 2:** Linear regression for renal elasticity, renal length, and kidney-to-liver elasticity ratio.

	Renal elasticity			Renal length			Kidney to Liver Elasticity ratio			
	β	95% CI	*P*-value	β	95% CI	*P*-value	Β	95% CI	*P*-value	
**Model 1**										
eGFR (ml/min/1.73 m^2^)	0.076	−0.055 to 0.206	0.255	0.184	0.148 to 0.220	<0.001	0.001	−0.001 to 0.003	0.214	
Log-transformed UPCR	−7.397	−10.082 to −4.711	<0.001	0.902	0.163 to 1.641	0.017	−0.136	−0.179 to −0.092	<0.001	
**Model 2**										
Age (yr)	0.127	−0.161 to 0.416	0.387	−0.218	−0.291 to −0.146	<0.001	0.001	−0.001 to 0.003	0.466	
female vs. male	3.199	−4.666 to 11.064	0.424	−4.138	−6.116 to −2.160	<0.001	−0.127	−0.171 to −0.084	<0.001	
DM	−0.989	−9.293 to 7.315	0.815	2.200	0.112 to 4.289	0.039	0.012	−0.006 to 0.031	0.193	
BMI	0.120	−1.040 to 1.280	0.839	0.426	0.134 to 0.718	0.004	−0.001	−0.005 to 0.004	0.777	
eGFR (ml/min/1.73 m^2^)	0.104	−0.045 to 0.253	0.171	0.151	0.114 to 0.189	<0.001	0.091	−0.034 to 0.217	0.154	
Log-transformed UPCR	−7.222	−9.952 to −4.492	<0.001	0.675	−0.012 to 1.361	0.054	−0.178	−0.310 to −0.045	0.009	
**Model 3**										
Age (yr)	0.130	−0.161 to 0.421	0.382	−0.231	−0.304 to −0.159	<0.001	−0.001	−0.005 to 0.004	0.774	
female vs. male	2.234	−5.990 to 10.458	0.594	−3.730	−5.770 to −1.690	<0.001	0.065	−0.066 to 0.196	0.329	
DM	−0.924	−9.335 to 7.486	0.829	1.711	−0.376 to 3.797	0.108	−0.179	−0.313 to −0.045	0.009	
BMI	0.210	−0.973 to 1.394	0.727	0.390	0.096 to 0.684	0.009	0.015	−0.004 to 0.034	0.125	
eGFR (ml/min/1.73 m^2^)	0.121	−0.034 to 0.276	0.126	0.146	0.108 to 0.185	<0.001	0.001	−0.001 to 0.004	0.283	
Log-transformed UPCR	−7.544	−10.469 to −4.619	<0.001	0.560	−0.166 to 1.286	0.130	−0.138	−0.184 to −0.091	<0.001	
Hemoglobin (g/dl)	−0.886	−3.052 to 1.280	0.422	0.345	−0.192 to 0.882	0.207	−0.024	−0.059 to 0.010	0.171	
Albumin (g/dl)	1.047	−6.248 to 8.342	0.778	−3.024	−4.834 to −1.214	0.001	0.012	−0.104 to 0.129	0.837	

UPCR: urine protein to creatinine ratio; DM: diabetes mellitus; BMI: body mass index; eGFR: estimated glomerular filtration rate.

**Table 3:** Logistic regression for rapid renal progression by renal elasticity, renal length, and kidney-to-liver elasticity ratio in patients with CKD stages 3-5.

	Renal elasticity (per 10 unit)		Renal length (per 1 mm)		Kidney to Liver Elasticity ratio (per 10%)	
	OR	*P*-value	OR	*P*-value	OR	*P*-value
Unadjusted	0.901 (0.844–0.962)	0.002	1.014 (0.993–1.035)	0.202	0.928 (0.885–0.923)	0.002
Model 1	0.901 (0.843–0.963)	0.002	1.013 (0.989–1.038)	0.295	0.926 (0.882–0.973)	0.002
Model 2	0.925 (0.862–0.992)	0.029	1.031 (1.004–1.059)	0.026	0.944 (0.897–0.993)	0.026
Model 3	0.928 (0.864–0.997)	0.042	1.022 (0.994–1.050)	0.125	0.942 (0.894–0.992)	0.030

Model 1 adjusts for age, sex, and diabetes.

Model 2 adjusts for covariates in model 1 plus eGFR and log-transformed UPCR.

Model 3 adjusts for covariates in model 2 plus BMI, hemoglobin and albumin.
