# Supplementary material for: Association of Renal Elasticity and Renal Function Progression in Patients with Chronic Kidney Disease Evaluated by Real-Time Ultrasound Elastography
Source: Sci Rep. 2017 Feb 27;7:43303. doi: 10.1038/srep43303 (PMC5327389; doi:10.1038/srep43303)

**Association of Renal Elasticity and Renal Function Progression in Patients with Chronic Kidney Disease Evaluated by Real-Time Ultrasound Elastography**

Hugo You-Hsien Lin^1,3,4,5,6,7^, Yu-Li Lee^2, 3^, Kun-Der Lin^2,3^, Yi-Wen Chiu^1^, Shyi–Jang Shin^2^, Shang-Jyh Hwang^1,4,5^, Hung-Chun Chen^1^, Chi-Chih Hung^1^

1. Division of Nephrology, ^2^Endocrinology and Metabolism, Department of Internal Medicine, Kaohsiung Medical University Hospital, Kaohsiung Medical University
2. Department of Internal Medicine, Kaohsiung Municipal Ta-Tung Hospital, Kaohsiung Medical University
3. Graduate Institute of Medicine, College of Medicine, Kaohsiung Medical University, Kaohsiung, Taiwan
4. Lipid Science and Aging Research Center, Kaohsiung Medical University, Kaohsiung
5. Department of Physiology & Biophysics, University of California at Irvine, Irvine, CA 92697, USA
6. Sue and Bill Gross Stem Cell Research Center, University of California at Irvine, Irvine, CA 92697, USA

Word count for the abstract: 198 words

Word count for the main text of the manuscript: 2860 words

Running title: Microscopic elastography in CKD

Corresponding author: Chi-Chih Hung

Division of Nephrology, Department of Internal Medicine, Kaohsiung Medical University Hospital, Kaohsiung Medical University

Telephone: 886-7-3121101 ext 7351-13

FAX: 886-7-3228721

E-mail: chichi@cc.kmu.edu.tw

Address: 100 Tzyou First Road, Kaohsiung 807, TAIWAN

**Supplementary Figure legends**

Figure 1 Tissue elasticity distribution was assessed in RTE by recording response to strain and stress within the regions of interest (ROI)

Supplementary Figure 1.


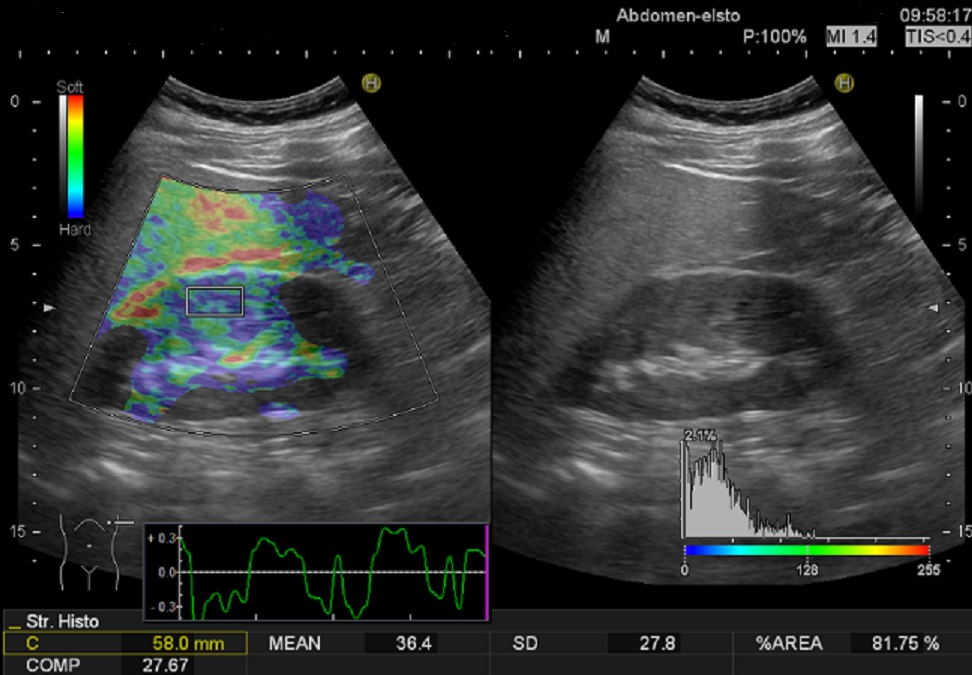

Supplement: Supplementary Figure [file srep43303-s1.doc]
